# A Novel Implant for Superior Pubic Ramus Fracture Fixation—Development and a Biomechanical Feasibility Study

**DOI:** 10.3390/medicina59040740

**Published:** 2023-04-10

**Authors:** Till Berk, Ivan Zderic, Jan Caspar, Peter Schwarzenberg, Torsten Pastor, Sascha Halvachizadeh, Biser Makelov, Geoff Richards, Hans-Christoph Pape, Boyko Gueorguiev

**Affiliations:** 1 AO Research Institute Davos, Clavadelerstrasse 8, 7270 Davos, Switzerland; 2 Department of Trauma, University Hospital Zurich, Raemistrasse 100, 8091 Zurich, Switzerland; 3 Department of Orthopaedic and Trauma Surgery, Cantonal Hospital Lucerne, 6000 Lucerne, Switzerland; 4 Harald-Tscherne Laboratory for Orthopedic and Trauma Research, University of Zurich, Sternwartstrasse 14, 8091 Zurich, Switzerland; 5 University Multiprofile Hospital for Active Treatment ‘Prof. Stoyan Kirkovitch’, Trakia University, 6003 Stara Zagora, Bulgaria

**Keywords:** experimental biomechanical approach, intramedullary splinting, novel fixation device, superior pubic ramus fractures, think outside the box in orthopedics

## Abstract

*Background and Objectives:* Pubic ramus fractures are common in compound pelvic injuries known to have an increased rate of morbidity and mortality along with recurrent and chronic pain, impeding a patient’s quality of life. The current standard treatment of these fractures is percutaneous screw fixation due to its reduced risk of blood loss and shorter surgery times. However, this is an intricate surgical technique associated with high failure rates of up to 15%, related to implant failure and loss of reduction. Therefore, the aim of this biomechanical feasibility study was to develop and test a novel intramedullary splinting implant for fixation of superior pubic ramus fractures (SPRF), and to evaluate its biomechanical viability in comparison with established fixation methods using conventional partially or fully threaded cannulated screws. *Materials and Methods:* A type II superior pubic ramus fracture according to the Nakatani classification was created in 18 composite hemi-pelvises via a vertical osteotomy with an additional osteotomy in the inferior pubic ramus to isolate the testing of three SPRF fixation techniques performed in 6 semi-pelvises each using either (1) a novel ramus intramedullary splint, (2) a partially threaded ramus screw, or (3) a fully threaded ramus screw. *Results:* No significant differences were detected among the fixation techniques in terms of initial construct stiffness and number of cycles to failure, *p* ≥ 0.213. *Conclusion:* The novel ramus intramedullary splint can be used as an alternative option for treatment of pubic ramus fractures and has the potential to decrease the rate of implant failures due to its minimally invasive implantation procedure.

## 1. Introduction

Pubic ramus fractures are commonly observed in compound pelvic injuries related to both high-energy and low-energy trauma [1]. They are among the most frequent fractures in trauma patients of all age groups and are knowingly associated with an increase in morbidity and mortality, recurrent and/or chronic pain, as well as impeded quality of life [2,3,4,5]. The overall incidence of pelvic ring fracture is 20–37/100,000 per year, and 92/100,000 per year for patients over 65 years of age [6,7]. Furthermore, the overall occurrence of pubic ramus fractures is 6.9/100,000 per year, and 25.6/100,000 per year for patients over 60 years of age [4]. The mortality could be linked to immobilization due to persistent/chronic pain. Percutaneous screw fixation is one of the standard procedures for treatment of superior pubic ramus fractures (SPRF), known for its reduced blood loss and shorter surgery times compared to plating [8,9,10]. However, percutaneous closed reduction and internal fixation (CRIF) in anterior pelvic ring injuries has been reported with failure rates of up to 15%, related to implant failure and loss of reduction [10,11,12]. Furthermore, minimally invasive screw fixation is known as an intricate surgical technique [13]. Alternative percutaneous surgical procedures for treatment of anterior pelvic ring fractures could therefore be of great benefit for this large anatomical region in the field of traumatology. An experienced and proven procedure from the early days of surgery is intramedullary splinting [14]. In the field of pediatrics, a notable increase in the use of elastic stable intramedullary nails—acting as splints for fracture treatment—was reported [15].

Biomechanical studies have concluded that the anterior structures of the pelvic ring provide only 40% of its stability and that the posterior sacroiliac (SI) complex accounts for the remaining 60% [16]. Therefore, a splinted surgical treatment of the anterior pelvic ring might be sufficient to ensure fracture healing and reasonable outcomes with potentially reduced surgery time and complications. Currently, there seems to be a gap in the literature regarding the splinting of pelvic fractures. Apart from a nail used for treatment of superior pubic ramus fractures, the authors are not aware of any comparable medical implant [13]. Therefore, the aim of this biomechanical feasibility study was to develop and test a novel intramedullary splinting implant for fixation of superior pubic ramus fractures, and to evaluate its biomechanical viability in comparison with established fixation methods using conventional partially or fully threaded cannulated screws.

## 2. Materials and Methods

### 2.1. Features of the Novel Intramedullary Splinting Implant

**Figure 1:**
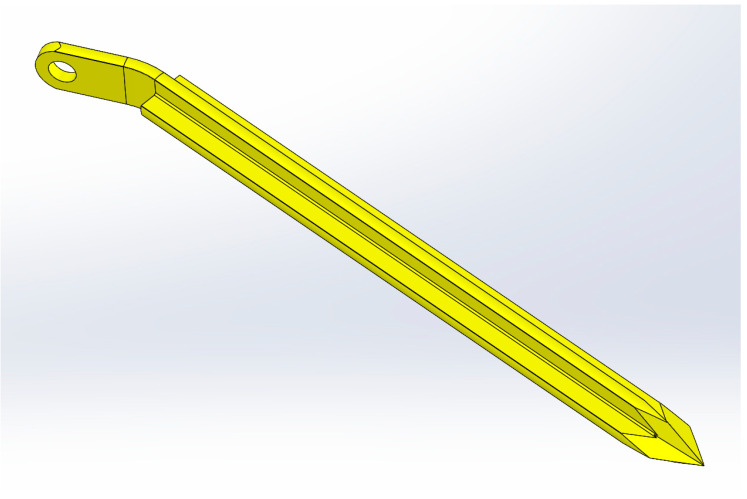
A 3D model of the novel intramedullary splinting implant for superior pubic ramus fracture fixation.

A three-dimensional (3D) model of the developed novel intramedullary splint for superior pubic ramus fracture fixation is visualized in Figure 1. Its principle relies on intramedullary nailing with an identical entry point as for retrograde intramedullary pubic ramus screws (pubic tubercle) [17]. The proximal part of the splint contains a flap which will rest flush on the outer cortex of the proximal region of the superior pubic ramus. The flap has a 4 mm hole accommodating one bicortical 3.5 mm screw to lock the splint in its final position. In addition, the splint has a cross-sectional shape consisting of four fins of equal length. The rigidity of the split within the intramedullary canal achieves stabilization of the fracture, while the four fins provide rotational control. The blunt advancement of the splint causes the hooking of the four fins in both the cortical bone at the entry point and the intramedullary spongiosa, thus ensuring that the fracture fragments are safeguarded against rotation. Additional rotational support is provided by the screw, which also prevents split’s migration away from the bone. A minimally invasive surgical technique, the same as the one performed for conventional ramus screw placement, is possible with the novel implant too. Due to the convenience of the method and the associated potentially shorter operation time, the novel implant could offer a significant advantage over the challenging standard treatment.

### 2.2. Specimens Preparation

Nine composite pelvises (LSS4060/Hard, Synbone, Zizers, Switzerland) were used. A superior pubic ramus fracture type II according to the Nakatani classification [10] was created by means of a vertical osteotomy within the middle zone of the superior pubic ramus, using a 1 mm bone sawblade and a custom template to ensure identical and reproducible fractures. In addition, the inferior pubic ramus was osteotomized to explicitly exclude its influence from a biomechanical point of view. Each pelvis was prepared for a separate testing of its left and right side, resulting in 18 available hemi-pelvises, assigned to 3 groups of 6 specimens each (n = 6), with equal number of right and left sides available for testing of different SPRF fixation methods using stainless steel implants as follows: Group Ramus Intramedullary Splint (RIS), using the novel splint (90 mm length, 7.3 mm width) with a 3.5 mm self-tapping screw (22 mm length, DePuy Synthes, Zuchwil, Switzerland); Group Ramus Screw Partial Threaded (RSP), using a 7.3 mm partially threaded cannulated screw (90 mm length, 32 mm partial thread, DePuy Synthes, Zuchwil, Switzerland); Group Ramus Screw Fully Threaded (RSF), using a 7.3 mm fully threaded cannulated screw (90 mm length, DePuy Synthes, Zuchwil, Switzerland).

**Figure 2:**
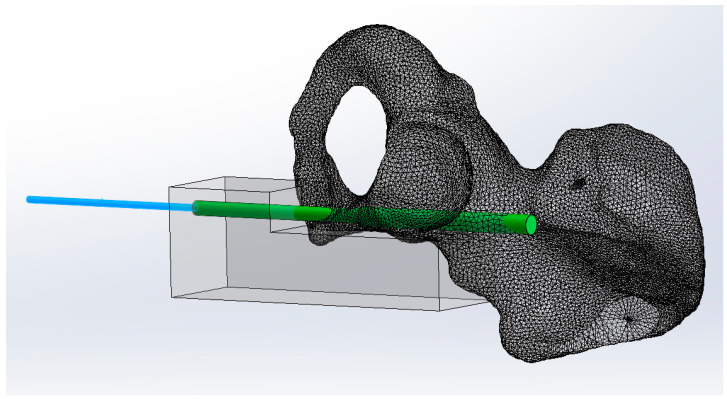
A custom 3D-printed aiming device for K-wire placement in the superior pubic ramus.

The SRPF fixation of the specimens was carried out according to the AO principles of fracture management [17]. An experienced surgeon with senior consultant status performed all procedures. In all groups, the fixation of the reduced SRPF commenced with the placement of a 2.8 mm Kirschner (K) guide wire in the superior pubic ramus in a retrograde fashion across the fracture and cephalad to the acetabulum. A custom-made 3D-printed aiming device was used to ensure standardized and repeatable wire and implant placements avoiding any perforations via falsa or cortical disruptions, while allowing the K-wire to be safely placed in the ideal position in one attempt (Figure 2).

**Figure 3:**
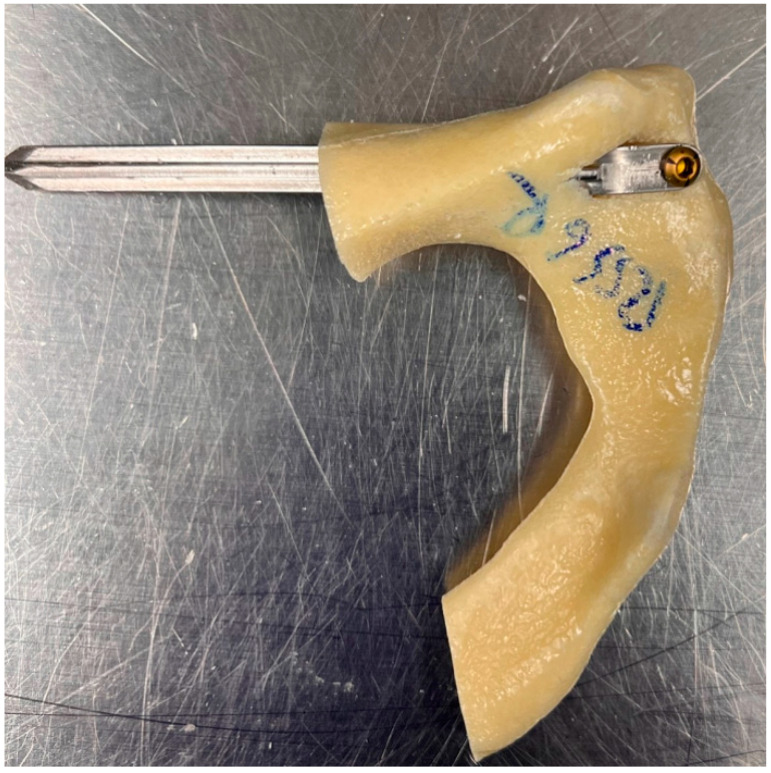
Intramedullary splinting implant in situ.

In the RIS group, a cannulated 6 mm drill was then used over the entire distance to be covered by the implant. The splint was inserted through the outer cortical drill hole by gently driving it forward with light hammer blows along the intramedullary canal across the fracture and cephalad to the acetabulum, spanning the fracture. The proximal flap of the splint, resting flush on the outer cortex, was locked in position with the screw after drilling a 2.5 mm pilot hole (Figure 3).

In the RSP and RSF groups, the starting point for screw insertion was the pubic tubercle, with a trajectory following the medial cortical boarder and avoiding the acetabulum.

**Figure 4:**
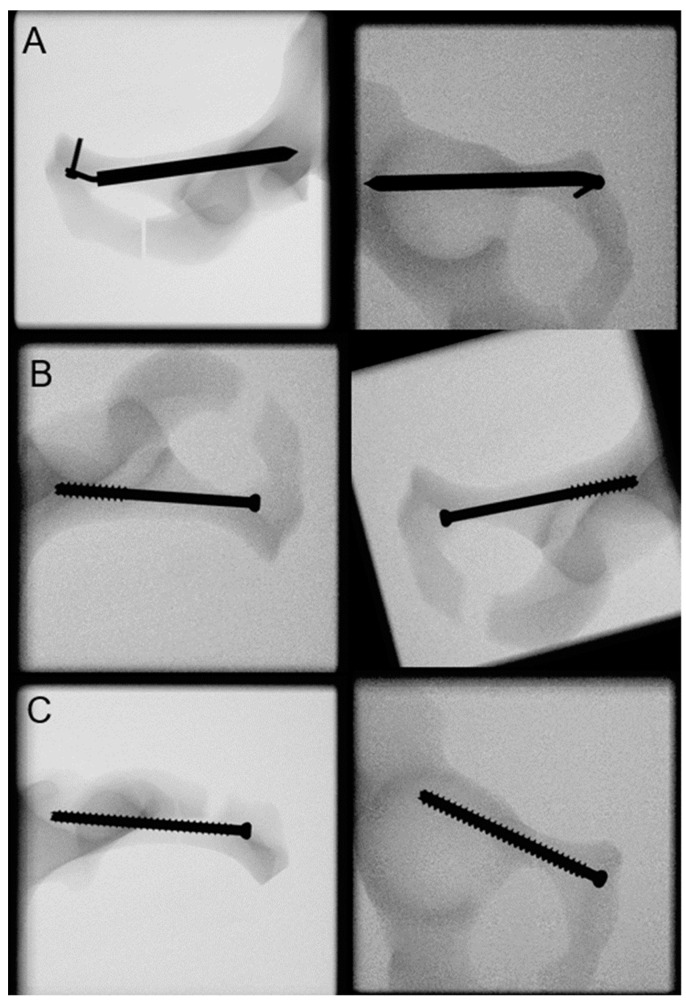
X-rays post instrumentation visualizing exemplified specimens from groups RIS (**A**), RSP (**B**), and RSF (**C**).

Inlet and obturator X-rays of all specimens were taken after instrumentation for documentation and verification of the implant positioning (Figure 4). In preparation for biomechanical testing, the sacroiliac joint aspect was embedded in a polymethylmethacrylate (PMMA, SCS-Beracryl D-28, Suter Kunststoffe AG/Swiss-Composite, Fraubrunnen, Switzerland) block. Finally, two retro-reflective marker sets were attached to the superior and inferior fragment adjacent to the fracture line for optical motion tracking.

### 2.3. Biomechanical Testing

**Figure 5:**
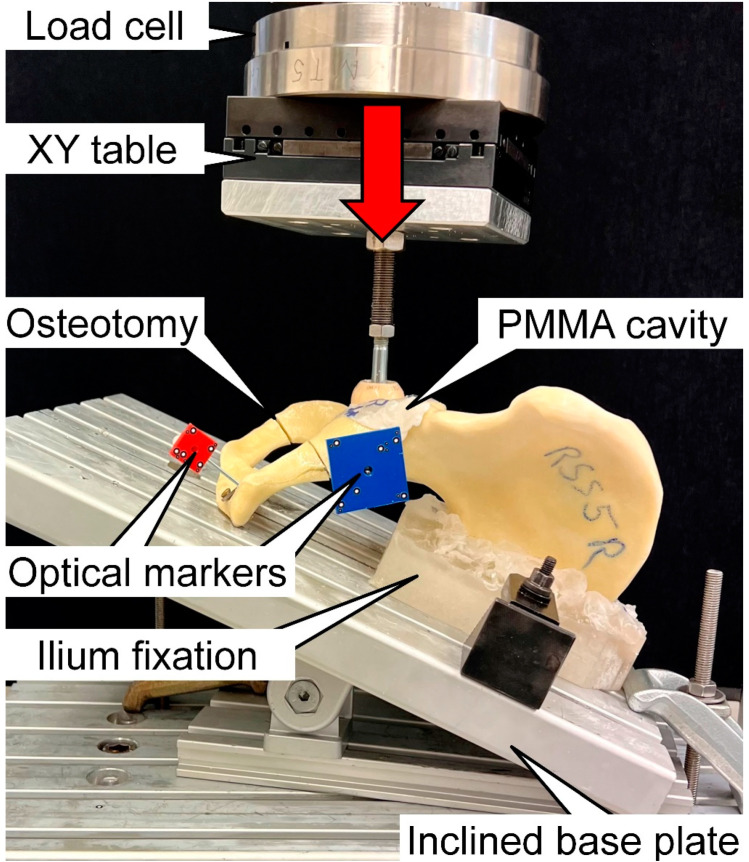
Setup with a specimen mounted for biomechanical testing and a vertical arrow denoting the loading direction.

Biomechanical testing was performed on a servohydraulic material test system (Mini Bionix II 858; MTS Systems, Eden Prairie, MN, USA) equipped with a 4 kN load cell (HUPPERT 6, HUPPERT GmbH, Herrenberg, Germany) using a setup adopted from a previous study [18] (Figure 5). Each hemi-pelvis was aligned and tested in an inverted upright standing position. For this purpose, it rested on an aluminum base plate, rigidly secured to the machine base and inclined at 20° in the coronal plane for positioning of both the medial aspect of the symphysis and the sacroiliac joint aspect flush with the base plate according to Morosato et al. [19]. The sacroiliac joint aspect was additionally constrained to the base plate.

Axial compression along the machine axis was applied to the acetabulum via a ceramic ball of 56 mm diameter. Homogenous load transfer to the specimens was achieved using a molded PMMA hemispherical cavity inserted in the acetabulum. This configuration targeted a simulation of a hip joint reaction force trajectory during walking, as previously described by Bergmann et al. and Jamari et al. [20,21]. The loading protocol commenced with a nondestructive quasi-static ramp from 20 N preload to 200 N at a rate of 18 N/s, followed by progressively increasing cyclic loading in axial compression with a physiological profile of each cycle at a rate of 2 Hz [20]. Keeping the valley load at a constant level of 20 N, the peak load, starting at 200 N, was monotonically increased cycle by cycle at a rate of 0.05 N/cycle until reaching a 10 mm actuator displacement with respect to its position at the beginning of the test. This test stop criterion was considered adequate to provoke catastrophic failure of all specimens [22,23].

### 2.4. Data Acquisition and Analysis

Axial displacement and axial load were continuously acquired throughout testing from the machine transducer and the load cell at 200 Hz, respectively. Based on these data, construct stiffness was calculated from the ascending load–displacement curve of the initial quasi-static ramp within the linear loading range between 80 N and 180 N. Furthermore, the coordinates of the optical markers attached to the specimens were continuously acquired throughout testing at 20 Hz by means of stereographic optical motion tracking using contactless full-field deformation technology (Aramis SRX, GOM GmbH, Braunschweig, Germany), operating at a resolution of 12 megapixel and maximum acceptance error of 0.004 mm to assess the interfragmentary movements in all six degrees of freedom. Based on the motion tracking data, the following parameters were evaluated: (1) displacement, defined as the magnitude of the relative interfragmentary translational movement between the two most anterior-superior pubic ramus aspects located on the two osteotomy planes of the simulated fracture; (2) gap angle, defined as the angle between the two osteotomy planes; and (3) torsional displacement, defined as the twisting interfragmentary angle around the implant axis. The outcome values of these parameters were analyzed after 2000, 4000, 6000, 8000, and 10,000 test cycles under peak loading with respect to the beginning of the cyclic test. The latter number represented the highest number of cycles when none of the specimens had failed so that dropouts could not artifactually affect the results.

Furthermore, 2 mm displacement, 5° gap angle, and 5° torsional displacement, relative to the initial position, were defined as clinically relevant failure criteria, and the corresponding numbers of cycles until fulfillment of each separate criterion under peak loading—defined as cycles to failure—were calculated together with their corresponding peak load, defined as failure load.

Statistical analysis was performed using SPSS software package (v.27, IBM SPSS, Armonk, NY, USA). Normality of data distribution was screened and proved with a Shapiro–Wilk test. Significant differences among the groups regarding stiffness, cycles to failure, and failure load were detected with One-Way Analysis of Variance (ANOVA) and Bonferroni post hoc test for multiple comparisons. General Linear Model Repeated Measures and Bonferroni post hoc tests were conducted to identify significant differences among the groups with regard to the parameters displacement, gap angle, and torsional displacement evaluated over the time points after 2000, 4000, 6000, 8000, and 10,000 cycles. The level of significance was set at 0.05 for all statistical tests.

## 3. Results

Construct stiffness (N/mm) was 305.5 ± 69.1 for RIS, 365.5 ± 111.1 for RSP, and 311.3 ± 80.5 for RSF, with no significant differences among them (*p* = 0.483).

**Figure 6:**
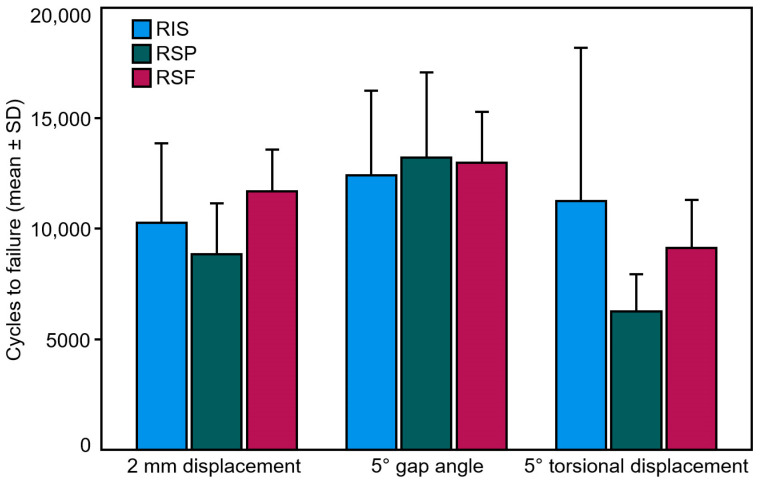
Cycles to failure in the study groups (RIS, RSP, and RSF) presented in terms of mean value and standard deviation according to the different failure criteria (2 mm displacement, 5° gap angle, and 5° torsional displacement).

Outcome measures of the parameters displacement, gap angle, and torsional displacement, analyzed over the five time points after 2000, 4000, 6000, 8000, and 10,000 cycles, are summarized in Table 1. No significant differences were detected among the groups with regard to each of these parameters (*p* ≥ 0.098). Displacement increased significantly over cycles in every separate group (*p* ≤ 0.045), whereas both gap angle and torsional displacement increased significantly over cycles only for RSF (*p* ≤ 0.006) but not for RIS and RSP (*p* ≥ 0.075). The numbers of cycles to failure and the corresponding failure loads according to the clinically relevant criteria (2 mm displacement, 5° gap angle, and 5° torsional displacement) are summarized in Table 2 and visualized in Figure 6. No significant differences were detected among the groups with regard to each of these two parameters (*p* ≥ 0.213). For RIS, the earliest failure occurred via reaching 5° gap angle in three specimens and 5° torsional displacement in three semi-pelvises, in contrast to RSP and RSF where it occurred unexceptionally via reaching 5° torsional displacement in all six specimens.

## 4. Discussion

A novel intramedullary splinting implant for SPRF fixation was developed and tested in this study, and its biomechanical viability was evaluated in comparison with established fixation methods using conventional partially or fully threaded cannulated screws. The novel implant has the potential to reduce implant failures, accommodate minimally invasive procedures, and provide comparable stability with convenient use versus standard fixation methods.

Due to the novelty of the fixation concept introduced in the present study, a comparison with previous published work was difficult. However, in one report, a similar splint for surgical treatment of rib fractures was described [24]. The supremacy of the rib splint versus K-wires was demonstrated in terms of rotational stability, eventually resulting in less slippage/collapse at the fracture site [25].

In a prospective randomized controlled trail, Andrade-Silva et al. compared intramedullary fixation to plating of midshaft clavicular fractures, reporting similar functional results, time to bone union, level of postoperative pain, and patient satisfaction rates, with a considerably expressed minimally invasive approach featuring the intramedullary fixation [26].

In a biomechanical cadaveric study investigating SPRF fixations, two 3.5 mm fully threaded screws were compared versus one 7.3 mm partially threaded screw to report comparable results regarding stability. The 3.5 mm screws were characterized as less demanding during insertion due to their deflection at the cortical margins [18]. The intramedullary splint in the current study is intended to function on the same principle. A clear advantage of the intramedullary splinting is the blunt implant advancement in the bone with a lower risk of bone perforation. The latter can be related to via falsa with a possible injury to the large nerves and vessels of the pelvis. In addition, the splint is guided in the bone and deflected away from the cortical margins with less demanding insertion, possibly resulting in reduced radiation exposure and operation time. Moreover, it can be used in the proximal part of the fracture as a joystick to achieve closed anatomical fracture reduction. Generally, this should also be possible with the guide wire used for cannulated screw placements, yet it would be much more difficult due to the significantly smaller wire diameter and the associated weaker lever arm. A closed joystick reposition via the cannulated screw itself does not seem possible due to the previously placed guide wire and the fixed position of the fracture.

Another related concept was presented in the literature, where a locking nail for treatment of SPRF was successfully tested clinically [13]. This underlines the claim of the currently introduced concept that a splint can be advanced bluntly in cancellous bone. However, the locking nail necessitates two screws for secure fixation, requiring at least one additional skin incision. Another possible advantage of our splint could be the possibility to deal with the entry point, implant advancement, and its proximal screw locking through the same initial incision. Furthermore, no additional instruments such as aiming devices or drill sleeves are needed. This highlights the simple and highly effective concept of the splint, which could also be very cost effective. The authors expect that this implant can be inserted in vivo without a guide wire and predrilling, which would fully exploit its advantages.

An anterior pelvic tilt of 13° on average has been reported previously [27]. This physiological positioning of the pelvis allows passive compression at the fracture site due to the smooth surface of the splint. However, active compression as with the use of a partially threaded screw cannot be achieved with the splint. An advantage of the fully threaded screw is the proximal anchoring of the thread in the bone, potentially providing more stability against screw migration. The splint offers the same advantage via its proximal anchoring through the 3.5 mm screw placement. Moreover, it rests flush on the proximal cortex with less prominent surface for potential irritation.

Pubic ramus fractures are still treated more often conservatively than operatively in the clinical practice, due to the known risk of chronic pain, long-term immobilization, and delayed functional outcome [28]. Grewal et al. state that there is a justifiable reluctancy towards percutaneous instrumentation of the structures at the front of the pelvis [29]. Finding a convenient and simple solution for an unpopular surgery technique was one of the main objectives of this study. The known drawbacks and difficulties of the existing techniques often force the treating surgeons to reject the fixation of anterior pelvic structures and choose a conservative treatment approach commanding long bed stays for the patient [30].

### Limitations

Even though the current study demonstrated comparable results of the intramedullary splint versus the current standard treatment option of minimally invasive screw fixation, no implant migration was observed in any of the tests. The authors interpret this as a possible limitation due to the use of artificial bones in this feasibility work. On the other hand, artificial bones allow for better standardization and homogenization of the specimens and procedures, thus principally overpowering the dissimilarities in the bone quality of human cadaveric specimens, with the latter also being less cost effective [31,32,33,34]. Moreover, synthetic bone specimens have been successfully used in various previous biomechanical studies, especially in ones focusing on the pelvis [31,35,36,37,38]. Furthermore, the availability of cadavers is restricted, leading to a very limited sample size implemented in biomechanical experimentations. Due to this, the sample size reported in previous work is relatively small [39]. While the use of standardized artificial bones minimizes the variability of test results between test specimens, the chosen sample size was still relatively small, yet comparable to equivalent biomechanical studies investigating pelvic fixation techniques [35,36,37,38,40]. Another possible drawback of the intramedullary splint could be the non-pronounced distal fixation, leaving room for possible movement at the fracture site. However, according to the AO principles, relative stability in this shaft-like area should be sufficient for uneventful fracture healing [41,42].

## 5. Conclusions

The novel intramedullary splinting implant developed in the current study demonstrates comparable stability versus standard implants used for fixation of superior pubic ramus fractures and can therefore be an alternative option for treatment in combination with the retrograde surgical approach. It has the potential to decrease the rate of implant failures due to its minimally invasive implantation procedure and could qualify as a straightforward surgical method potentially exposing the patient to less radiation. Further studies are necessary to pave the way for its use in the clinical practice. The hypothesis of blunt advancement of the splint in the cancellous bone would be of particular interest. Furthermore, the required operation time and radiation exposure in comparison to the standard screw fixation should be the subject of a future investigation.

## Figures and Tables

**Table 1:** Outcome measures of the parameters displacement, gap angle, and torsional displacement evaluated over the five time points after 2000, 4000, 6000, 8000, and 10,000 cycles, presented for each separate group (RIS, RSP, and RSF) in terms of mean value and standard deviation, together with *p*-values from the statistical comparisons among the groups (*p* *) and over cycles (*p* **).

Parameter	Group	Cycles					*p* *
		2000	4000	6000	8000	10,000	
Displacement [mm]	RIS	0.29 ± 0.11	0.59 ± 0.23	1.11 ± 0.52	2.02 ± 1.35	2.39 ± 1.30	0.098
	*p* **	0.015					
	RSP	0.41 ± 0.21	0.78 ± 0.31	1.26 ± 0.41	1.78 ± 0.59	2.88 ± 1.49	
	*p* **	0.045					
	RSF	0.16 ± 0.07	0.37 ± 0.14	0.66 ± 0.26	0.99 ± 0.38	1.40 ± 0.46	
	*p* **	<0.001					
Gap angle [°]	RIS	0.26 ± 0.22	0.71 ± 0.41	1.40 ± 0.84	3.10 ± 3.32	3.93 ± 3.46	0.569
	*p* **	0.075					
	RSP	0.36 ± 0.21	0.64 ± 0.41	1.00 ± 0.44	1.36 ± 0.53	2.83 ± 2.36	
	*p* **	0.164					
	RSF	0.37 ± 0.23	0.64 ± 0.40	1.08 ± 0.61	1.66 ± 0.79	2.45 ± 1.41	
	*p* **	0.006					
Torsional displacement [°]	RIS	0.93 ± 0.49	1.72 ± 1.05	3.56 ± 2.74	6.94 ± 7.74	7.94 ± 7.91	0.412
	*p* **	0.092					
	RSP	1.66 ± 0.83	3.09 ± 1.18	4.85 ± 1.27	7.25 ± 2.22	11.17 ± 6.74	
	*p* **	0.081					
	RSF	0.53 ± 0.25	1.30 ± 0.59	2.65 ± 1.46	4.22 ± 2.28	6.32 ± 2.96	
	*p* **	0.004					

**Table 2:** Cycles to failure and failure loads until fulfillment of the clinically relevant failure criteria (2 mm displacement, 5° gap angle, and 5° torsional displacement), presented for each separate group (RIS, RSP, and RSF) in terms of mean value and standard deviation, together with *p*-values from the statistical comparisons among the groups (*p*).

Criterion	Group	Failure		*p*
		Cycles	Load [N]	
2 mm displacement	RIS	10,279 ± 3590	1227.9 ± 359.0	0.257
	RSP	8860 ± 2302	1086.0 ± 230.2	
	RSF	11,703 ± 1882	1370.3 ± 188.2	
5° gap angle	RIS	12,414 ± 3835	1441.4 ± 383.5	0.918
	RSP	13,220 ± 3859	1522.0 ± 385.9	
	RSF	13,000 ± 2292	1500.0 ± 229.2	
5° torsional displacement	RIS	14,901 ± 4413	1690.1 ± 441.3	0.213
	RSP	6254 ± 1686	825.4 ± 168.6	
	RSF	9145 ± 2163	1114.5 ± 216.3	

## Data Availability

The datasets used and/or analyzed during the current study are available from the corresponding author on reasonable request.

## References

[1] Rommens P.M., Hopf J.C., Herteleer M., Devlieger B., Hofmann A., Wagner D. (2020). Isolated pubic ramus fractures are serious adverse events for elderly persons: An observational study on 138 patients with fragility fractures of the pelvis type I (FFP type I). J. Clin. Med..

[2] Scheyerer M.J., Osterhoff G., Wehrle S., Wanner G.A., Simmen H.P., Werner C.M. (2012). Detection of posterior pelvic injuries in fractures of the pubic rami. Injury.

[3] Van Dijk W., Poeze M., Van Helden S.H., Brink P.R.G., Verbruggen J.P.A.M. (2010). Ten-year mortality among hospitalised patients with fractures of the pubic rami. Inj. Int. J. Care Inj..

[4] Hill R.M.F., Robinson C.M., Keating J.F. (2001). Fractures of the pubic rami: Epidemiology and five-year survival. J. Bone Jt. Surg..

[5] Breuil V., Roux C.H., Testa J., Albert C., Chassang M., Brocq O., Euller-Ziegler L. (2008). Outcome of osteoporotic pelvic fractures: An underestimated severity. Survey of 60 cases. Jt. Bone Spine.

[6] Melton L.J., Sampson J.M., Morrey B.F., Ilstrup D.M. (1981). Epidemiologic features of pelvic fractures. Clin. Orthop. Relat. Res..

[7] Kannus P., Palvanen M., Niemi S., Parkkari J., Järvinen M. (2000). Epidemiology of osteoporotic pelvic fractures in elderly people in Finland: Sharp increase in 1970–1997 and alarming projections for the new millennium. Osteoporos. Int..

[8] Li C. (2014). Clinical comparative analysis on unstable pelvic fractures in the treatment with percutaneous sacroiliac screws and sacroiliac joint anterior plate fixation. Eur. Rev. Med. Pharmacol. Sci..

[9] Gire J.D., Jiang S.Y., Gardner M.J., Bishop J.A. (2018). Percutaneous versus open treatment of posterior pelvic ring injuries: Changes in practice patterns over time. J. Orthop. Trauma.

[10] Starr A.J., Nakatani T., Reinert C.M., Cederberg K. (2008). Superior pubic ramus fractures fixed with percutaneous screws: What predicts fixation failure?. J. Orthop. Trauma.

[11] Routt M.C., Nork S.E., Mills W.J. (2000). Percutaneous fixation of pelvic ring disruptions. Clin. Orthop. Relat. Res..

[12] Routt M.C., Simonian P.T., Mills W.J. (1997). Iliosacral screw fixation: Early complications of the percutaneous technique. J. Orthop. Trauma.

[13] Ivanov P.A., Zadneprovsky N.N., Nevedrov A.V., Kalensky V.O. (2018). Pubic Rami Fractures Fixation by Interlocking Intramedually Nail: First Clinical Experience. Traumatol. Orthop. Russ..

[14] Kuentscher G. Intramedullary Splinting—a Brief History. Proceedings of the Ciba Symposium.

[15] Tarng Y.W., Lin K.C., Chen C.F., Yang M.Y., Chien Y. (2021). The elastic stable intramedullary nails as an alternative treatment for adult humeral shaft fractures. J. Chin. Med. Assoc..

[16] Tile M. (1996). Acute pelvic fractures: I. Causation and classification. JAAOS J. Am. Acad. Orthop. Surg..

[17] Nambiar M., West L.R., Bingham R. (2017). AO Surgery Reference: A comprehensive guide for management of fractures. Br. J. Sport. Med..

[18] Acklin Y.P., Zderic I., Grechenig S., Richards R.G., Schmitz P., Gueorguiev B. (2017). Are two retrograde 3.5 mm screws superior to one 7.3 mm screw for anterior pelvic ring fixation in bones with low bone mineral density?. Bone Jt. Res..

[19] Morosato F., Traina F., Cristofolini L. (2018). Standardization of hemipelvis alignment for in vitro biomechanical testing. J. Orthop. Res..

[20] Bergmann G., Deuretzbacher G., Heller M., Graichen F., Rohlmann A., Strauss J., Duda G.N. (2001). Hip contact forces and gait patterns from routine activities. J. Biomech..

[21] Jamari J., Ammarullah M.I., Santoso G., Sugiharto S., Supriyono T., Permana M.S., Winarni T.I., van der Heide E. (2022). Adopted walking condition for computational simulation approach on bearing of hip joint prosthesis: Review over the past 30 years. Heliyon.

[22] Gueorguiev B., Ockert B., Schwieger K., Wähnert D., Lawson-Smith M., Windolf M., Stoffel K. (2011). Angular stability potentially permits fewer locking screws compared with conventional locking in intramedullary nailed distal tibia fractures: A biomechanical study. J. Orthop. Trauma.

[23] Windolf M., Muths R., Braunstein V., Gueorguiev B., Hänni M., Schwieger K. (2009). Quantification of cancellous bone-compaction due to DHS® Blade insertion and influence upon cut-out resistance. Clin. Biomech..

[24] Marasco S., Saxena P. (2015). Surgical rib fixation–Technical aspects. Injury.

[25] Bottlang M., Helzel I., Long W., Fitzpatrick D., Madey S. (2010). Less-invasive stabilization of rib fractures by intramedullary fixation: A biomechanical evaluation. J. Trauma Acute Care Surg..

[26] Andrade-Silva F.B., Kojima K.E., Joeris A., Silva J.S., Mattar R. (2015). Single, superiorly placed reconstruction plate compared with flexible intramedullary nailing for midshaft clavicular fractures: A prospective, randomized controlled trial. JBJS.

[27] Vialle R.A., Levassor N.I., Rillardon L.U., Templier A.L., Skalli W.A., Guigui P.I. (2005). Radiographic analysis of the sagittal alignment and balance of the spine in asymptomatic subjects. J. Bone Jt. Surg..

[28] Solomon L.B., Pohl A.P., Sukthankar A., Chehade M.J. (2009). The subcristal pelvic external fixator: Technique, results, and rationale. J. Orthop. Trauma.

[29] Grewal I.S., Starr A.J. (2020). What’s New in Percutaneous Pelvis Fracture Surgery?. Orthop. Clin..

[30] Ghanayem A.J., Wilber J.H., Lieberman J.M., Motta A.O. (1995). The effect of laparotomy and external fixator stabilization on pelvic volume in an unstable pelvic injury. J. Trauma Acute Care Surg..

[31] Gardner M.P., Chong A.C., Pollock A.G., Wooley P.H. (2010). Mechanical evaluation of large-size fourth-generation composite femur and tibia models. Ann. Biomed. Eng..

[32] Heiner A.D. (2008). Structural properties of fourth-generation composite femurs and tibias. J. Biomech..

[33] Zdero R., Olsen M., Bougherara H., Schemitsch E.H. (2008). Cancellous bone screw purchase: A comparison of synthetic femurs, human femurs, and finite element analysis. Proc. Inst. Mech. Eng. Part H J. Eng. Med..

[34] Elfar J., Stanbury S., Menorca R.M.G., Reed J.D. (2014). Composite bone models in orthopaedic surgery research and education. J. Am. Acad. Orthop. Surg..

[35] Camino Willhuber G., Zderic I., Gras F., Wahl D., Sancineto C., Barla J., Windolf M., Richards R.G., Gueorguiev B. (2016). Analysis of sacro-iliac joint screw fixation: Does quality of reduction and screw orientation influence joint stability? A biomechanical study. Int. Orthop..

[36] Gardner M.J., Kendoff D., Ostermeier S., Citak M., Hüfner T., Krettek C., Nork S.E. (2007). Sacroiliac joint compression using an anterior pelvic compressor: A mechanical study in synthetic bone. J. Orthop Trauma.

[37] Yinger K., Scalise J., Olson S.A., Bay B.K., Finkemeier C.G. (2003). Biomechanical comparison of posterior pelvic ring fixation. J. Orthop. Trauma.

[38] Sahin O., Demirors H., Akgun R., Tuncay I. (2013). Internal fixation of bilateral sacroiliac dislocation with transiliac locked plate: A biomechanical study on pelvic models. Acta Orthop. Traumatol. Turc..

[39] Sagi H., Ordway N., DiPasquale T. (2004). Biomechanical analysis of fixation for vertically unstable sacroiliac dislocations with iliosacral screws and symphyseal plating. J. Orthop. Trauma.

[40] Van Zwienen C.M., van den Bosch E.W., van Dijke G.A.H., Snijders C.J., van Vugt A.B. (2005). Cyclic loading of sacroiliac screws in tile C pelvic fractures. J. Trauma Inj. Infect. Crit. Care.

[41] Perren S.M. (1989). The biomechanics and biology of internal fixation using plates and nails. Orthopedics.

[42] McKibbin B. (1978). The biology of fracture healing in long bones. J. Bone Jt. Surg..

